# Descriptive analysis of dietary (poly)phenol intake in the subcohort MAX from DCH-NG: “Diet, Cancer and Health—Next Generations cohort”

**DOI:** 10.1007/s00394-022-02977-x

**Published:** 2022-08-22

**Authors:** Fabian Lanuza, Raul Zamora-Ros, Agnetha Linn Rostgaard-Hansen, Anne Tjønneland, Rikard Landberg, Jytte Halkjær, Cristina Andres-Lacueva

**Affiliations:** 1 Biomarkers and Nutrimetabolomics Laboratory, Department of Nutrition, Food Sciences, and Gastronomy, Food Innovation Network (XIA), Institute of Nutrition and Food Safety (INSA), Faculty of Pharmacy and Food Sciences, University of Barcelona, 08028 Barcelona, Spain; 2 CIBER of Frailty and Healthy Aging (CIBERFES), Instituto de Salud Carlos III, 28029 Madrid, Spain; 3 Unit of Nutrition and Cancer, Cancer Epidemiology Research Program, Catalan Institute of Oncology (ICO), Bellvitge Biomedical Research Institute (IDIBELL), Gran Via de L’Hospitalet, 199, L’Hospitalet de Llobregat, 08908 Barcelona, Spain; 4 Danish Cancer Society Research Center, Strandboulevarden 49, DK 2100 Copenhagen, Denmark; 5 Department of Biology and Biological Engineering, Division of Food and Nutrition Science, Chalmers University of Technology, Gothenburg, Sweden

**Keywords:** Phenolic compounds, Dietary intake, Food sources, Meals, Denmark

## Abstract

**Purpose:**

(Poly)phenols are bioactive compounds widely distributed in plant-based foods. Currently, limited data exist on the intake distribution of (poly)phenols across meals. This study aimed to estimate dietary intakes of all individual (poly)phenols and total intake per class and subclass by meal event, and to identify their main food sources in the subcohort MAX from the Diet, Cancer and Health—Next Generations cohort (DCH-NG).

**Methods:**

Dietary data were collected using three web-based 24-h dietary recalls over 1 year. In total, 676 participants completed at least one recall. The dietary data were linked to Phenol-Explorer database using standardized procedures and an in-house software. We categorized foods/drinks into five options of meal events selected by the participant: 'Breakfast', 'Lunch', 'Evening', 'Snack', and 'Drink'.

**Results:**

Adjusted total (poly)phenols mean intake by meal was the highest in the drink event (563 mg/day in men and 423 mg/day in women) and the lowest in the evening event (146 mg/day in men and 137 mg/day in women). The main overall (poly)phenol class contributor was phenolic acids (55.7–79.0%), except for evening and snack events where it was flavonoids (45.5–60%). The most consumed (poly)phenol subclasses were hydroxycinnamic acids and proanthocyanidins. Nonalcoholic beverages (coffee accounted for 66.4%), cocoa products, and cereals were the main food sources of total (poly)phenols.

**Conclusion:**

This study provides data on the variability in the intake of classes and subclasses of (poly)phenols and their main food sources by meal event according to lifestyle data, age, and gender in a Danish population.

**Supplementary Information:**

The online version contains supplementary material available at 10.1007/s00394-022-02977-x.

## Introduction

(Poly)phenols are natural compounds that can range from simple molecules to highly polymerized structures with at least two phenolic groups attached to one or several benzene-rings [1]. In food, they commonly occur in conjugated forms, with one or more sugars linked to hydroxyl groups [2]. Phenolic compounds are classified based on their chemical structure into four main classes: flavonoids, phenolic acids, lignans, and stilbenes [3]. Flavonoids and phenolic acids are the main contributing classes of (poly)phenols in human diets. The principal food sources are fruits, vegetables, whole-grain cereals, cocoa, and beverages such as coffee, tea and wine [4].

(Poly)phenol intake is difficult to estimate accurately in epidemiologic studies for several reasons. First, the (poly)phenol content in food and drinks varies according to factors such as environmental conditions, genetics, food chain industry stages, among others. Second, databases on (poly)phenol content in foods have specific methodological limitations, ranging from different analytical methods used for the analysis of (poly)phenols to differences in classification of the phenolic compounds. Moreover, (poly)phenol databases are typically restricted to a limited number of available food items, and data on specific (poly)phenols in particular foods may be lacking [5]. To overcome some of these limitations and to improve the estimation of the (poly)phenol intake, there is a need for a unified system combining databases and published literature as well as the generation of more original food composition data on (poly)phenols and the update of the databases [6].

Furthermore, there is no gold standard approach for estimating (poly)phenol intake [5]. There are, however, strategies and procedures for standardizing the calculations of dietary (poly)phenol intake that take into consideration recipes, cooking methods, and food processing [7]. The most common methods for the assessment of usual diet in epidemiological studies are multiple 24-h dietary recalls (24-HDR) and food frequency questionnaires (FFQs). Such methods have been linked to (poly)phenol food composition databases to allow intake estimations in various populations worldwide, but particularly in Europe and America [4, 8].

The literature shows that sociodemographic and lifestyle risk factors such as age group, education, physical activity, alcohol and tobacco consumption, nutritional status, and others influence food and (poly)phenol intakes [9–11]. Irrespective of such confounders, there is growing evidence from epidemiological studies that (poly)phenol intake is associated with a reduction in chronic diseases and all-cause mortality [12].

The study of diets involves individual and collective eating behavior, associated with culture, education, tendencies, food security and globalization [13, 14]. For example, the implementation of healthy food environment policies could improve the consumption of (poly)phenol-rich foods. Moreover, a strong influence of Western diets and processed foods is commonly observed [15]. Family food-related dynamics may play an elemental role in dietary patterns, feeding behavior, and thereof also in (poly)phenol intake [16, 17]. Typically a meal event refers to the occasion or certain time that foods or drinks are consumed during the day, currently, meal events or menus in (poly)phenol estimation studies are scarce and their investigation could be interesting for analyzing [18].

We aimed to estimate the dietary (poly)phenol intake and the variability there of in a Danish subcohort of the “Diet, Cancer and Health—Next Generation” (DCH—NG) MAX study. In addition, we also studied the main food sources of (poly)phenols and their classes, subclasses and the most individual (poly)phenols consumed. All estimates were focused on a meal event perspective.

## Methods

### Study population

This study was a subsample from the Danish population-based DCH-NG cohort that was initiated in August 2015 and ended in April 2019. The DCH-NG cohort consists of 39,554 individuals aged between 18 and 79 who were biological children (generation 2), their spouses (generation 2-A) or grandchildren (generation 3) of the participants (generation 1) from the DCH cohort [19]. The objective of the establishment of the DCH-NG cohort was to be able to investigate associations between genes, diet, and lifestyle across generations. A total of 183,764 persons were invited to participate in the DCH-NG cohort [19]. This DCH-NG was a branch of the DCH prospective cohort that has been characterized previously [20].

A validation subcohort called “MAX” was conducted with 720 participants enrolled from August 2017 until January 2019 was used for the present study. To validate a semi-quantitative food frequency questionnaire and analyze long-term reproducibility of plasma and urine metabolites, among other aims, a subsample of the participants from the DCH-NG cohort was invited to participate in the MAX study. Thus, the MAX sample is not representative of the general population because the cohort participants can be considered a selected population as a previously mentioned [19]. The data and samples were collected at baseline, 6 and 12 months. Participants completed two main questionnaires concerning lifestyle and food frequency, 24-HDRs and participated in a health examination including collection of biological samples as well as anthropometric measurements and blood pressure measurements. We excluded the participants who did not have any 24-HDR, 676 individuals were included in this analysis.

The Diet, Cancer and Health—Next Generations research project was approved by the Danish Data Protection Agency ((journal number 2013–41–2043/2014–231–0094) and by the Committee on Health Research Ethics for the Capital Region of Denmark (journal number H-15001257). All participants provided their written informed consent to participate in the study.

### (Poly)phenol dietary intake

Participants in the DCH-NG MAX study filled out two 24-HDR (*n* = 676) at each time point, i.e. at baseline (*n* = 648), 6 (*n* = 406) and 12 months (*n* = 382), using a validated web-based tool myfood24 (www.myfood24.org) from Leeds University [21], which has been linked primarily with the Danish national food database and now contains approximately 1600 Danish food items, including a recipe maker. At each time point, one recall was completed in the day before and one recall in the day of examination. The participants reported all food consumed the day before the examination at the study center in grams by total portion size (as specified/selected by each participant). To calculate the (poly)phenol dietary intake, the mean of total (poly)phenols was used from the recall of the days before the examination because it was complete and represented a typical day. Also, the 24-HDR in the day of examination do not resemble a full day, since participants have been fasting from 1 to more than 9 h, respectively. Moreover, it was possible to add dietary supplements, and before finishing the recall a list of food items often forgotten was shown automatically by the myfood24 tool. Finally, portion sizes was based on reports from the Danish Food Institute.

Regarding processed foods, such as industrially or pre-packaged meals, the (poly)phenol estimation was made according to the percentages of ingredients in the food products. The complex food products were calculated as recipes taking into account the individual ingredients and their corresponding proportions as estimated from standardized recipes or data available on the Internet [22], especially from Danish websites. In complementary, we used recipes from the FFQ of the DCH cohort [23]. The overall procedure to link the reported food items followed the stepwise protocol reported by Knaze et al. [7]. A specific protocol was worked out to estimate the dietary intake of (poly)phenols from DCH-NG MAX dietary recalls using an “in-house” software developed by the University of Barcelona, the Bellvitge Biomedical Research Institute (IDIBELL) and the Centro de Investigation Biomédica en Red (CIBER) [18]. The first step was to prepare an ingredient list for each food item and highlight those with (poly)phenol content from the dietary recalls. The second step was to create the links between 24-HDR and the Phenol-Explorer database [24]. The third step was to calculate the total and individual (poly)phenols by food, meal and day.

We categorized foods/drinks into five options of meal events selected by the participant: 'Breakfast', 'Lunch', 'Evening', 'Snack', and 'Drink'. It is important to mention that the meal events were assigned by each participant according to their interpretation of the meal events, without a previous definition by the researchers. This could lead them to assign the same food item to different meal events, especially for drinks. For example, the breakfast, lunch, evening and snack events consider a 13.4%, 2.3%, 4.3%, and 10.5% of drinks in the 24HDRs, respectively. The total (poly)phenol content by meal event was calculated as the sum of all individual compounds expressed as they are found in food items (i.e. glycosides, aglycones, and esters) using the Phenol-Explorer database and in-house databases. In addition, the total dietary intake of (poly)phenols was presented by flavonoids (subclasses), phenolic acids, lignans, stilbenes and other (poly)phenols, and the most consumed (poly)phenols for each (poly)phenol class and subclass by meal, and their food sources. The data used in the present study were mainly acquired by chromatography without previous hydrolysis of the food extracts. Also, proanthocyanidin (PA) dimer data were obtained by chromatography without hydrolysis; however, for PA with a polymerization degree higher than two [PA trimers, PA 4–6 mers, PA 7–10 mers, and PA polymers (> 10 mers)], data obtained by normal-phase HPLC were used.

Data were missing from food items such as grapeseed, safflower and peanut oil, pumpkin seeds, tea rooibos hibiscus, and some alcohol drinks. Some missing values were replaced from similar foods, for example using botanical family and plant part. In dishes or recipes that used cooked oil, we used a standard proportion of oil absorption, i.e. 5% for stir-fried, 15% for fried, and 25% for deep-fried [25]. The (poly)phenol content of dehydrated or concentrated foods was estimated by multiplying the (poly)phenol contents of “normal” food items by a concentration factor., For example, the (poly)phenol content of expresso, a type of concentrated coffee, was calculated by multiplying the content of traditional filtered coffee by 2.0 [4]. Similarly, the concentration factors applied were: 3.0 for potato flour from raw potato, 0.5 for cocoa powder of cocoa solids from chocolate products, and 0.2 for jam or marmalade from raw fruit. In cereals and grains, we converted the weight change factor between dry and cooked weight, which was 3.8 for brown rice, 2.5 for pasta, rice and lentils, 2.4 for beans, and 2.1 for chickpeas. Lastly, we used different sources of traditional conversion weights for fresh and dried herbs. In the present study, the use of retention factors was not considered because they do not have a relevant impact on the estimated dietary (poly)phenols [4].

### Statistical analysis

All data were calculated with the mean intake (based on either one, two or three 24-HDRs) of days for total or meal events by an individual. Dietary (poly)phenol intakes according to meal events were estimated using general linear models and presented as adjusted means and standard errors (s.e.). Differences in (poly)phenol intakes for meals stratified by time origin, sex, age (18–34, 35–50 and > 50y), BMI (< 25, 25–30 and > 30 kg/m^2^), smoking status (never, former, and current smoker), and physical activity (regular vs no regular exercise) were compared using general linear models. All these models were adjusted for sex, age (year), time origin, total energy intake (kcal/day), and BMI (kg/m^2^) (as appropriate). *P* values < 0.05 (two-tailed) were considered significant.

The contribution of each (poly)phenol class and subclass to the total (poly)phenol intake was calculated as a percentage according to the five meal events: breakfast, lunch, evening, snack, and drink. The top 30 most consumed individual (poly)phenols and their main food sources in total and sex-specific population were calculated as a means and standard errors (s.e.) All analyses were conducted using SPSS software (IBM SPSS Statistics, version 27.0).

## Results

**Fig. 1:**
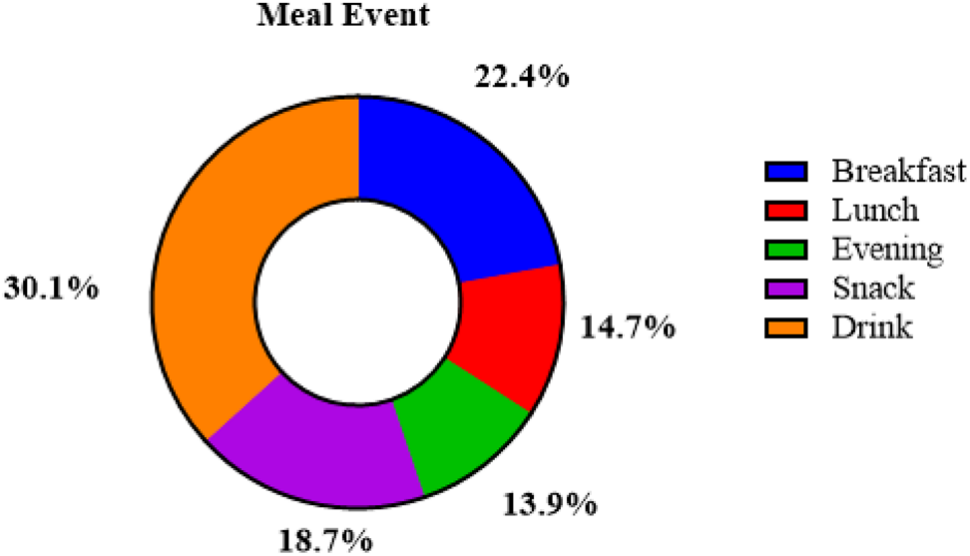
Percentage of total (poly)phenol mean content from meal events in all sample (*n* = 676)

The percentages of total (poly)phenol dietary intakes according to meal events are shown in Fig. 1. Among different meal events, drink events were the highest contributor (30.1%), while the lowest contributor to total (poly)phenol intake was evening (13.9%). Mean intakes of unadjusted and adjusted total (poly)phenols by meal adjusted for sex, age (y), time origin, BMI, and total energy intake are shown in Supplementary Table 1 and in Table 1, respectively. For the drink event, the total (poly)phenol intake was higher in men than in women, but the opposite was observed for the snack event. The intake of total (poly)phenols increased with age, especially at breakfast and with the drink event. The total (poly)phenol intake at both lunch and the drink event was higher in obese subjects. The total (poly)phenol intake at breakfast and at lunch showed a higher nonsignificant trend at the extremes of BMI (< 25 or > 30 kg/m2), compared with individuals with 25–30 kg/m2. When comparing the intake by smoking status, the drink event had the highest intake of total (poly)phenols as observed in current smokers (602 mg/day), followed by former smokers (530 mg/day) and never smokers (432 mg/day). In all meal events, (poly)phenol intake was higher with higher energy consumption, with the exception of the drinks event. Alcohol drinkers consumed more total (poly)phenols than nonconsumers, the contribution of alcoholic beverages to the overall (poly)phenols intake in alcohol drinkers was 7.0% (4.8% for wine). No differences in total (poly)phenol intake were observed in terms of time origin and physical activity. Moreover, no differences were observed in total polyphenols and by classes in all participants according to seasons (Supplementary Table 2).

**Table 1:** Adjusted^a^ mean daily intakes of total and (poly)phenols meal event by sex, age, and lifestyle factors in MAX subcohort

Stratification variable	*N*	All (mg/day)			Breakfast (mg/day)			Lunch (mg/day)			Evening (mg/day)			Snack (mg/day)			Drink (mg/day)		
		Mean	s.e	*p* value	Mean	s.e	*p* value	Mean	s.e	*p* value	Mean	s.e	*p* value	Mean	s.e	*p* value	Mean	s.e	*p* value
Total (poly)phenols	676	1325	34		293	12		159	7		141	5		245	11		486	26	
Time origin				0.380			0.396			0.743			0.229			0.100			0.196
Baseline	648	1406	39		316	16		180	9		144	7		317	17		846	48	
6 months	406	1319	50		350	20		169	11		162	9		288	23		727	62	
12 months	382	1392	51		320	21		174	11		141	9		359	24		875	64	
Sex				0.272			0.184			0.716			0.405			0.050			0.008
Men	305	1366	49		274	18		162	10		146	8		219	17		563	38	
Women	371	1292	44		308	16		156	9		137	7		266	15		423	35	
Age (year)				< 0.001			0.006			0.234			0.494			0.174			< 0.001
18–34	197	1081	62		229	23		149	13		145	10		209	21		347	48	
35–50	210	1350	58		305	22		177	12		132	9		249	20		485	45	
> 50	269	1495	52		326	19		152	11		147	8		262	18		607	40	
Body mass index (kg/m^2^)				0.109			0.087			0.049			0.943			0.408			0.049
< 25	371	1317	45		310	17		163	9		140	7		257	15		446	35	
25–30	236	1292	55		254	21		141	12		144	9		227	19		524	43	
> 30	69	1531	102		324	38		202	22		138	17		219	36		646	80	
Smoking status				0.144			0.819			0.663			0.407			0.348			0.025
Never smoker	353	1275	45		284	17		153	9		147	7		257	15		432	35	
Former smoker	186	1352	64		296	24		165	13		141	10		218	22		530	50	
Current smoker	137	1438	72		303	27		167	15		127	12		236	25		602	56	
Physical activity				0.425			0.821			0.841			0.454			0.949			0.509
Regular	562	1317	35		290	13		158	7		140	6		242	12		485	28	
Not regular	114	1388	81		297	30		162	17		151	13		244	28		532	63	
Energy intake (kcal)				< 0.001			< 0.001			< 0.001			< 0.001			< 0.001			0.315
Tertile 1	225	1083	60		235	23		133	13		114	10		116	21		483	48	
Tertile 2	226	1371	63		302	24		164	13		135	11		210	21		558	50	
Tertile 3	225	1679	60		352	23		207	13		173	10		370	20		576	48	
Alcohol intake (g/day)				0.017			0.103			0.185			0.151			0.169			0.339
No	339	1307	51		277	19		160	11		133	8		219	18		515	40	
Yes	337	1466	54		318	20		179	11		150	9		252	19		566	43	

^a^Means and standard error (s.e.) were computed using general linear models adjusted for sex, age, total energy intake, and body mass index (as appropriate). *p* value is for differences in means according to the models

Phenolic acids were the main contributors to total (poly)phenols at breakfast, lunch, and drinks events (55.7–79.0%). In contrast, flavonoids were the main contributors at evening and snack events (45.5–60.0%). Lignans and stilbenes accounted for < 1.0% of total (poly)phenol intake. Other (poly)phenols accounted for 4.7% of total (poly)phenols, with the alkylphenols subclass being especially relevant (2.9%). However, the other (poly)phenols and alkylphenols were higher at the lunch event with 14.2% and 11.3%, respectively (Table 2).

**Table 2:** Percentage contribution of classes and subclasses of total (poly)phenols, and the top three most consumed polyphenols for each polyphenol class and subclass by meal in MAX study

Polyphenol classes and subclasses	All (%)	Breakfast (%)	Lunch (%)	Evening (%)	Snack (%)	Drink (%)	Top three most consumed individual (poly)phenols
Total (poly)phenols	100	100	100	100	100	100	5-Caffeoylquinic acid; 4-caffeoylquinic acid; 3-caffeoylquinic acid
Flavonoids	31.8	24.4	26.7	45.5	60.0	19.6	Proanthocianidins: polymers (> 10 mers); 04–06 mers; 07–10 mers
Anthocyanins	1.9	1.8	1.3	3.8	1.5	1.7	Malvidin 3-*O*-glucoside; cyanidin 3-*O*-glucoside; cyanidin 3-*O*-rutinoside
Chalcones	< 0.1	0.0	< 0.1	< 0.1	< 0.1	< 0.1	Xanthohumol
Dihydrochalcones	0.1	0.2	0.1	0.1	0.3	< 0.1	Phloridzin; phloretin 2'-*O*-xylosyl-glucoside; 3-hydroxyphloretin 2'-*O*-glucoside
Dihydroflavonols	0.2	< 0.1	< 0.1	0.5	< 0.1	0.3	Dihydromyricetin 3-*O*-rhamnoside; dihydroquercetin 3-*O*-rhamnoside
Flavanols	23.4	16.4	16.3	24.5	54.9	13.8	Proanthocianidins: polymers (> 10 mers); 04–06 mers; 07–10 mers
Flavan-3-ol monomers	5.6	4.3	2.6	2.8	5.6	7.7	(-)-Epicatechin; (-)-epigallocatechin 3-*O*-gallate; (-)-epigallocatechin
Proanthocyanidins	17.4	11.5	13.6	21.6	49.1	5.3	Proanthocianidins: polymers (> 10 mers); 04–06 mers; 07–10 mers
Theaflavins	0.4	0.6	0.1	0.1	0.2	0.8	Theaflavin 3'-*O*-gallate; theaflavin 3,3'-*O*-digallate; theaflavin
Flavanones	0.7	1.3	0.6	0.7	0.3	0.7	Hesperidin; didymin; narirutin
Flavones	1.8	2.6	3.9	4.3	1.2	0.2	Apigenin 6,8-C-galactoside-C-arabinoside; apigenin 6,8-C-arabinoside-C-glucoside; apigenin 6,8-di-C-glucoside
Flavonols	3.5	2.0	4.2	11.1	1.8	3.0	Quercetin 3-*O*-rutinoside; quercetin 3,4'-*O*-diglucoside; kaempferol 3-O-glucoside
Isoflavonoids	0.1	< 0.1	0.3	0.6	< 0.1	< 0.1	6''-*O*-Malonylgenistin; 6''-*O*-Malonyldaidzin; Genistin
Phenolic acids	62.6	69.7	55.7	42.4	37.5	79.0	5-Caffeoylquinic acid; 4-caffeoylquinic acid; 3-caffeoylquinic acid
Hydroxybenzoic acids	2.2	2.2	2.3	2.9	2.2	1.9	5-*O*-Galloylquinic acid; gallic acid; sanguiin H-6
Hydroxycinnamic acids	60.3	67.5	53.2	39.2	35.2	77.0	5-Caffeoylquinic acid; 4-caffeoylquinic acid; 3-caffeoylquinic acid
Hydroxyphenylacetic acids	< 0.1	< 0.1	0.1	0.2	< 0.1	< 0.1	Homovanillic acid; 4-hydroxyphenylacetic acid; methoxyphenylacetic acid
Hydroxyphenylpropanoic acids	< 0.1	0.0	< 0.1	< 0.1	< 0.1	0.0	Dihydro-p-coumaric acid; dihydrocaffeic acid
Stilbenes	0.1	< 0.1	< 0.1	< 0.1	< 0.1	0.1	Piceatannol 3-*O*-glucoside; resveratrol 3-*O*-glucoside; d-viniferin
Lignans	0.8	1.0	3.3	1.4	0.3	< 0.1	Secoisolariciresinol; lariciresinol; pinoresinol
Other (poly)phenol class	4.7	4.8	14.2	10.6	2.0	1.2	5-Nonadecylresorcinol; 5-heneicosylresorcinol; 5-heptadecylresorcinol
Alkylphenols	2.9	3.5	11.3	4.3	1.2	0.1	5-Nonadecylresorcinol; 5-heneicosylresorcinol; 5-heptadecylresorcinol
Tyrosols	0.8	0.1	1.8	4.4	0.3	0.2	3,4-DHPEA-EDA; p-HPEA-EDA; tyrosol
Alkylmethoxyphenols	0.3	0.3	0.3	0.4	0.1	0.3	4-Ethylguaiacol; 4-vinylguaiacol; 4-vinylsyringol
Curcuminoids	0.2	0.4	0.4	0.9	< 0.1	0.0	Curcumin; demethoxycurcumin; bisdemethoxycurcumin
Other (poly)phenol subclass	0.5	0.5	0.4	0.6	0.4	0.6	Pyrogallol; catechol; phlorin

With regard to (poly)phenol subclasses, the most important contributors to total (poly)phenol intake were hydroxycinnamic acids (60.3%), ranging from 39.2% at the evening event to 77% at the drinks event. Flavanols were the main contributors in snacks (54.9%) and the second-highest contributors in all other meal events, especially in the form of the proanthocyanidins oligomers and polymers. They were followed by flavonols (2.0–11.1%), alkylphenols (0.1–11.3%), hydroxybenzoic acids (1.9–2.9%), and anthocyanins (1.3–3.8%), as well as flavones, flavanones, and tyrosol, each of which accounted for 0.1–4.4% of meal events. The rest of the subclasses were less remarkable, each one contributing less than 1% to total (poly)phenol intake and meal events. Overall, the drinks event presents a great difference in subclasses of (poly)phenols such as flavanols, flavones, hydroxycinnamic acids, and alkylphenols compared to other meal events.

**Fig. 2:**
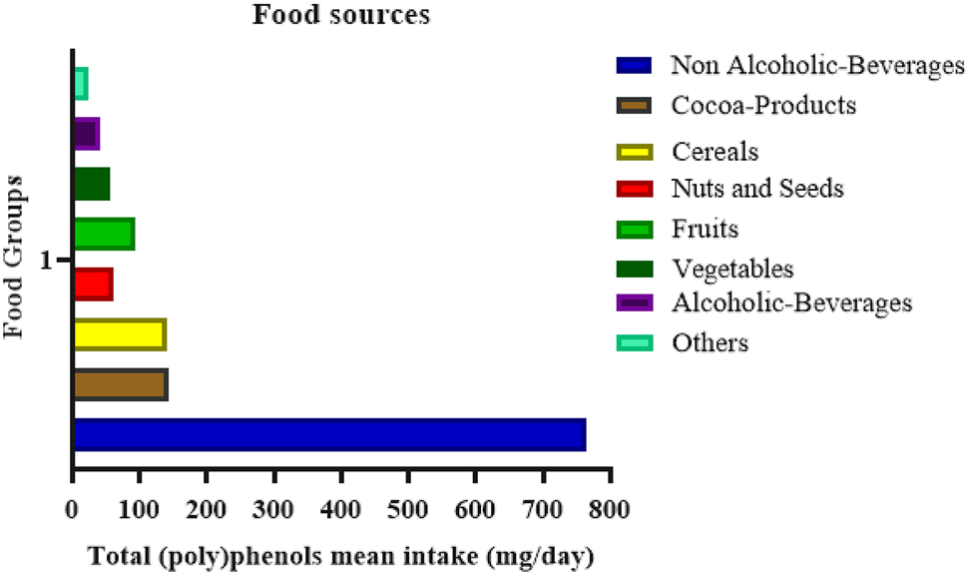
Total (poly)phenol mean intake from main food groups in all sample (*n* = 676). The (poly)phenol contribution is expressed as amount in mg/day

**Fig. 3:**
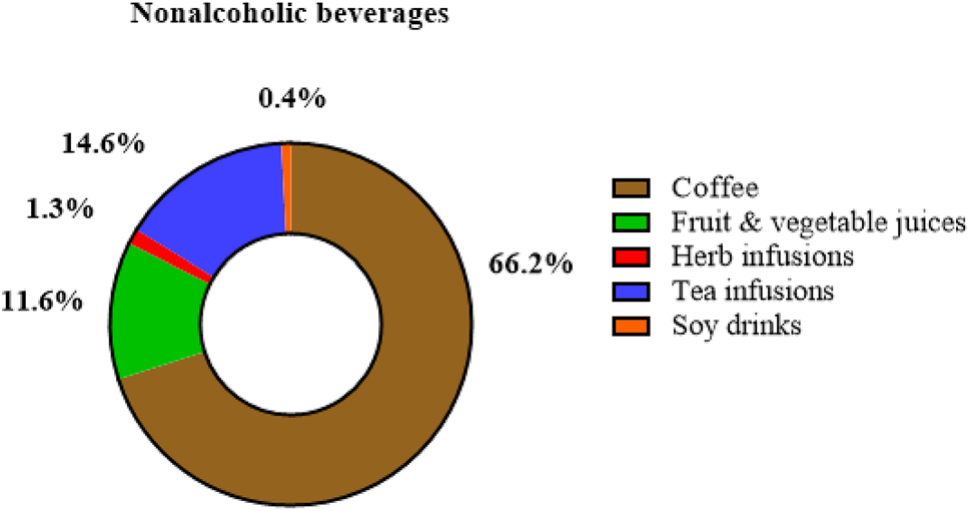
Total mean percentage distribution of (poly)phenols from nonalcoholic beverages in all sample (*n* = 676). The (poly)phenol contribution is expressed as amount in mg/day

The main food sources of total (poly)phenols are presented in Fig. 2. Nonalcoholic beverages (764 mg/day), cocoa products (144 mg/day), and cereals (140 mg/day) were the major dietary sources of total (poly)phenols. Food sources that have an intermediate contribution were nuts and seeds, and fruits and vegetables, ranging from 55 mg/day to 94 mg/day. Alcoholic beverages and other food sources such as oils and herbs were lower contributors with means of 41 mg/day and 24 mg/day, respectively. Coffee (66.4%) and tea infusions (14.6%) were the most important nonalcoholic beverages contributors (Fig. 3).

The mean intake of the top 30 individual (poly)phenols is shown in Table 3 together with their five main food sources. The top three most consumed (poly)phenols were three caffeoylquinic acid subtypes, where the largest food source was coffee (82.5–99.2%). The 5-caffeoylquinic acid intake in the total sample was 272 mg/day, with the intake being similar in women (274 mg/day) and in men (268 mg/day). They were followed by proanthocyanidin polymers (61 mg/day) and proanthocyanidin 4–6 oligomers (37 mg/day), while cocoa products, nuts, and apples were the main food sources. Ferulic acid (50 mg/day) had a high consumption, particularly coming from cereals, especially rye bread (57.8%). Tea consumption was the main food source of (−)-epigallocatechin 3-*O*-gallate, (−)-epigallocatechin, ( +)-gallocatechin, and 5-*O*-galloylquinic acid (> 90%). Finally, cereal products like rye bread were an important source of alkylphenols such as 5-nonadecylresorcinol, 5-heneicosylresorcinol, and 5-heptadecylresorcinol.

**Table 3:** Mean intakes (mg/day) of top 30 most consumed individual (poly)phenols and their main food sources in MAX study

	(Poly)phenol (mg/day)	(Poly)phenol Subclass	All mean (s.e.)	Men mean (s.e.)	Women mean (s.e.)	Top five main food sources
1	5-Caffeoylquinic acid	Hydroxycinnamic acids	272 (8.2)	268 (13.4)	274 (10.1)	Coffee (82.5%); potatoes (5.9%); seeds (3.5%); apples (1.4%); blueberries (1.0%)
2	4-Caffeoylquinic acid	Hydroxycinnamic acids	181 (6.8)	181 (11.3)	182 (8.3)	Coffee (99.2%); tea (0.35%); tomatoes (0.16%); apples (0.08%); stone fruits (0.02%)
3	3-Caffeoylquinic acid	Hydroxycinnamic acids	151 (5.9)	152 (9.8)	151 (7.2)	Coffee (98.0%); stone fruits (1.1%); broccoli (0.4%); tea (0.2%); wine rose (0.1%)
4	Proanthocyanidin polymers (> 10 mers)	Flavanols	104 (4.0)	113 (5.5)	96 (5.8)	Cocoa products (41.5%); nuts and pulses (14.6%); apples (8.8%); wine and grapes (6.4%); berries (5.1%)
5	Ferulic acid	Hydroxycinnamic acids	69 (1.8)	67 (2.8)	70 (2.4)	Rye bread (57.8%); corn and corn products (8.1); wheat bread (7.8%); breakfast cereals (4.2%); pastry (4.1%)
6	Proanthocyanidin 4–6 oligomers	Flavanols	65 (2.5)	70 (3.3)	60 (3.7)	Cocoa products (46.6%); apple (12.8%); almond (5.5%); wine and grapes (4.7%); beans (3.0%)
7	Proanthocyanidin 7–10 oligomers	Flavanols	43 (1.6)	46 (2.1)	40 (2.3)	Cocoa products (41.8%); apples (18.0%); almond (7.0%); wine and grapes (5.6%); beans (5.2%)
8	5-Feruloylquinic acid	Hydroxycinnamic acids	40 (1.4)	40 (2.3)	41 (1.7)	Coffee (99.6%); Root vegetables (0.3%); Blueberries (0.05%)
9	4-Feruloylquinic acid	Hydroxycinnamic acids	38 (1.3)	37 (2.0)	39 (1.8)	Coffee (99.8%); Root vegetables (0.15%); Fruits (0.05%)
10	Procyanidin dimer B2	Flavanols	27 (0.8)	29 (1.3)	26 (1.1)	Cocoa products (25.7%); tea (25.3); apples (16.1%); nuts and seeds (5.7%); wine (5.5%)
11	5-*O*-Galloylquinic acid	Hydroxybenzoic acids	27 (1.8)	27 (2.7)	26 (2.5)	Tea (99.9%); berries (0.1%)
12	3,4-Dicaffeoylquinic acid	Hydroxycinnamic acids	24 (0.7)	24 (1.2)	25 (1.0)	Coffee (95.5%); root vegetables (4.5%)
13	Proanthocyanidin trimers	Flavanols	20 (0.7)	22 (1.1)	19 (0.9)	Cocoa products (37.4%); apple (23.0%); nuts and seeds (7.6%); wine (4.8%); tea (3.7%)
14	4,5-Dicaffeoylquinic acid	Hydroxycinnamic acids	19 (0.6)	19 (1.0)	19 (0.7)	Coffee (100%)
15	(−)-Epicatechin	Flavanols	17 (0.5)	18 (0.8)	17 (0.8)	Cocoa products (30.6%); Tea (28.7%); apples (18.4%); wine (9.3%); fruits (3.5%)
16	3,5-Dicaffeoylquinic acid	Hydroxycinnamic acids	16 (0.5)	16 (0.7)	17 (0.6)	Coffee (90.5%); root vegetables (9.5%)
17	(−)-Epigallocatechin 3-*O*-gallate	Flavanols	15 (1.1)	16 (1.8)	15 (1.5)	Tea (99.4%); herbal tea (0.4%); avocado (0.1%); nuts (0.05%)
18	Apigenin 6,8-C-galactoside-C-arabinoside	Flavones	14 (0.5)	14 (0.6)	13 (0.7)	Wheat bread (75.2%); pastry (8.8%); pasta and pizza (7.4%)
19	3-Feruloylquinic acid	Hydroxycinnamic acids	13 (0.5)	13 (0.8)	13 (0.6)	Coffee (99.1%); root vegetables (0.6%); stone fruits (0.2%)
20	5-Nonadecylresorcinol	Alkylphenols	11 (0.3)	11 (0.4)	11 (0.4)	Rye bread (71.5%); wheat bread (15.4); breakfast cereals (5.7%); pastry (3.4%); pasta & pizza (1.4%)
21	(−)-Epigallocatechin	Flavanols	11 (0.8)	11 (1.3)	10 (1.1)	Tea (97.9); nuts (1.4%); wine (0.2%); herbal tea (0.1%)
22	( +)-Catechin	Flavanols	11 (0.3)	11 (0.4)	10 (0.5)	Cocoa products (26.0%); wine (25.9%); tea (19.4%); fruits (12%)
23	5-Heneicosylresorcinol	Alkylphenols	10 (0.2)	10 (0.4)	10 (0.3)	Rye bread (64.8); wheat bread (10.9%); breakfast cereals (8.8%); pastry (5.2%); pasta and pizza (3.6%)
24	( +)-Gallocatechin	Flavanols	9 (0.7)	9 (1.0)	9 (1.0)	Tea (99.5%); wine (0.4%)
25	Apigenin 6,8-C-arabinoside-C-glucoside	Flavones	9 (0.3)	9 (0.4)	9 (0.5)	Wheat bread (72.5%); pastry (9.7%); pasta and pizza (7.8%)
26	Secoisolariciresinol	Lignans	8 (0.3)	8 (0.5)	9 (0.5)	Bread with seeds (66%); bread (23.5%); flaxseeds (7.6%); nonalcoholic beverages (0.6%); carrot (0.3%)
27	(−)-Epicatechin 3-*O*-gallate	Flavanols	8 (0.5)	8 (0.7)	8 (0.6)	Tea (87%); herbal tea (8.8%); wine (3.7%); fruits (0.3%)
28	Trans-Ferulic acid	Hydroxycinnamic acids	8 (0.5)	8 (0.6)	8 (0.7)	Bread (26.7%); breakfast cereal (17.2%); pasta, pizza, and rice (18.2%); corn chips (14%)
29	Quercetin 3-*O*-rutinoside	Flavonols	7 (0.4)	7 (0.7)	6 (0.5)	Tea (74.4%); wine (4.9%); stone fruits (4.9%); asparagus (3.4%)
30	5-Heptadecylresorcinol	Alkylphenols	6 (0.2)	6 (0.2)	7 (0.2)	Bread (90.9%); breakfast cereal (6.5%); pasta (1.2%)

## Discussion

Dietary intake of total (poly)phenols by meal events, (poly)phenol classes and subclasses, and individual (poly)phenols were estimated across the MAX subcohort from Denmark, using three 24-HDRs over 1 year, the Phenol-Explorer database, and an in-house protocol and software. The main food sources were also identified and the influence of sociodemographic and lifestyle factors on (poly)phenol estimation by meal events was also assessed. In recent years, the USDA and Phenol-Explorer databases have been commonly used for estimating individual or total (poly)phenols separately or in a customized way [12]. Phenol-Explorer allowed us to include more classes and subclasses of (poly)phenols, and express them as they are found in nature, mainly as glycosides and esters [26].

When comparing our total (poly)phenols (1325 mg/day) with other similar studies using Phenol-Explorer, comparable mean intakes (1193 mg/day) were estimated by six 24-HDRs in the SUVIMAX study [27]. Moreover, the EPIC study showed 1284 mg/day in the non-Mediterranean region, which included Denmark, using a single 24-HDR [4]. The PREDIMED study estimated a lower mean intake of 820 mg/day, in this case using a FFQ with 137 food items [28] and a Polish study with an intake of 989 mg/day by a single 24-HDR [29]. The HAPIEE study presented a higher mean of 1740 mg/day by a FFQ with 148 food items. Moreover, Latin American studies revealed lower intakes than European countries, thus, median intakes of 377–694 mg/day of total (poly)phenols were reported in a Brazilian and Mexican study, respectively [9, 30]. However, another Brazilian study using multiple 24-HDRs estimated a higher median intake of 1102 mg/day. There are definitely important differences between estimations of dietary (poly)phenols that can be explained by population and regional characteristics, such as food availability and food consumption culture, and methodological reasons, such as type of dietary questionnaire and the selected food composition database.

To the best of our knowledge, there are no large epidemiological studies that have estimated dietary (poly)phenol intake by meal events. In a randomized controlled trial study in older subjects, combining the Phenol-Explorer and USDA databases, [18] the control diet group showed a higher proportion of (poly)phenols for lunch and dinner events and a lower proportion for snacks compared to the (poly)phenol-rich diet group. These results can be explained by the (poly)phenol-rich diet used, in which (poly)phenols-rich products were used, especially in the snack meal event. Nevertheless, the net estimation on meals is higher in the (poly)phenol-rich diet. If these findings are compared with our results, the proportion of total (poly)phenol consumed by meal events was higher at breakfast (293 vs. 173 mg/day), lower at lunch (159 vs. 173 mg/day), and similar at dinner (141 vs. 140 mg/day) when compared to the (poly)phenol-rich diet. However, it is important to mention that the meal distribution was different because they did not have the drink event and some (poly)phenol-rich products used in the intervention were classified into the snack meal event [18]. Another recent study found that lunch is the meal with the highest intake of total (poly)phenols (mainly from coffee and cocoa products) in adult women, but this was estimated using Phenol-Explorer and the Folin–Ciocalteu method [31]. Our results suggest that the Danish population consumed more (poly)phenol-rich foods during breakfast, snacks, and drink events than at both lunch and evening events. Finally, getting information on meal events has interesting implications because it gives possibilities to develop strategies for increasing and spreading (poly)phenol intake over the day, which may be preferable to ensure a high and stable concentration throughout the entire day [32].

Hydroxycinnamic acids are by far the largest contributors to the total (poly)phenol intake due to the high coffee consumption (mean intake of 649 mg/day), which explains the 77.8% of the phenolic acid intake, being slightly higher than that observed in other studies [33–35]. It is important to mention that diluted filtered coffee is what is mostly consumed in Denmark, and the type of coffee is very important, because in some countries coffee is prepared in a concentrated form as in espresso, which is two- and fourfold richer in (poly)phenols than normal filtered coffee and filtered diluted coffee (American coffee), respectively [9]. Another popular beverage that largely contributes to total (poly)phenols, and specifically to flavonoids (20.7%) and phenolic acids (1.5%), is tea. When comparing the consumption of the same tea (mean intake of 96.1 mg/day) with other countries from the Mediterranean region (4.6%), in our study it was higher (7.3%), but it was lower than in Poland (27%) and the UK (40.8%) where the consumption is greater [33, 35]. The main contributor to hydroxybenzoic acids and a number of the most consumed individual (poly)phenols was tea, as found in other studies; however, in the PREDIMED study, it mainly came from olives and red wine in accordance with the food preferences in Mediterranean countries [28, 33]. Finally, thearubigins were not included in our analysis due to limitations presented previously in the EPIC study [36]. Furthermore, (poly)phenol intake data must be carefully compared because thearubigins could make a substantial contribution [37].

In the present study, the total (poly)phenol contribution of proanthocyanidin (17.4%) and flavonol (3.5%) subclasses from cocoa products, fruits and nuts and seeds is also worth noting. This is consistent with other studies [33, 38]. In the case of cocoa products (e.g. chocolate bar, chocolate shake milks, bonbons), the percentage of cocoa powder is important to be quantified because it provides the main/total part of the (poly)phenols in the whole product. It is important to bear in mind the existing differences in the (poly)phenol content in cocoa products between databases [39, 40]. The intake of whole grain in Denmark over the last decade has become very relevant, especially from rye and whole meal bread [41]. The significant contribution of lignans and alkylphenols to total (poly)phenols was higher in the present study, which focused on breakfast, than in other studies [33], with 3.3–11.3%, respectively. The contribution in other countries was mainly from refined wheat flour products and breakfast cereals [9, 27]. However, the phenolic data from whole-grain foods in Phenol Explorer is partially incomplete, which may cause an underestimation of their contribution.

When compared with main food sources in our study, the French SUVIMAX study estimated a lower mean intake of 658 mg/day from nonalcoholic beverages (coffee represented 79% of the total). The differences are largest in the PREDIMED study in which only 192 mg/day of total (poly)phenols were provided by nonalcoholic beverages (55% from coffee). Interestingly, the EPIC study presented a comprehensive comparison of prevalence in food consumption between Mediterranean and non-Mediterranean countries, highlighting large differences in nonalcoholic beverages (42.3 vs 62.6%, respectively), especially coffee and tea. While, Polish studies showed the highest intake of nonalcoholic beverages with 1150 mg/day (44% from coffee and 27% from tea) and 743.6 mg/day by 24-HDR [29, 35]. One study compared the mean total dietary (poly)phenol intake in coffee consumers (984 mg/day) and noncoffee consumers (456 mg/day) [42]. On the other hand, these studies showed a lower proportion of (poly)phenols from cocoa products and cereals but a higher proportion from fruits than in our results [27, 28, 33]. Certainly, food sources are closely related to the region and food tradition of the population, as typically occurs in the Mediterranean diet as well as in other countries or regions such as the Nordic diet from Scandinavian. For instance, in our study, the low amount of (poly)phenols at lunch and evening compared to snacks can be explained by the food choices of the MAX participants. (Poly)phenol-rich foods such as beverages (tea, coffee), fruits, and cocoa products were typically consumed in snack events, while vegetables were more consumed during the main meals. The content of (poly)phenols per portion size is lower in vegetables compare to fruits, tea, coffee, and cocoa products. The snack meal could be a good strategy to increase the dietary (poly)phenol intake [43]. Thus, one should be aware and consider the impact of dietary (poly)phenol content when comparing these food sources and their intakes, as well as the implications for studies and variables or outcomes. The potential limitations of questionnaires (type, administration form, design, data collection, etc.) applied by the study design should also be borne in mind.

In general, several studies have demonstrated that young people have a significantly lower (poly)phenol consumption than older people [12, 38, 44]. However, the (poly)phenol intake tends to increase or decrease in relation to age, and this can vary by population for many reasons, including eating habits, lifestyles, and seasons, among other things [45–47]. Indeed, some populations have demonstrated certain exceptions by subclasses [33]. Associations between (poly)phenol intake and lifestyle factors have been examined in several studies [33, 48]. A recent study showed an inverse association between flavonoids and mortality in smokers and consumers of high levels of alcohol, which should encourage more effort and the use of dietary interventions with a view to preventing cardiovascular diseases [11]. Also, current smokers consumed more total (poly)phenols and phenolic acids, as smokers are much more likely to drink coffee [9], which is consistent with our results. The effect of (poly)phenol intake on overweight/obesity is still unclear. The current literature supports an association between higher flavonoid intake and decreased body weight [10, 49]. Furthermore, commonly obese individuals follow high-caloric diets, unhealthy lifestyles and food habits, and that may alter (poly)phenol bioavailability and compromise the metabolism pathways more [50]. Finally, future studies with a prospective design and randomized controlled trials are needed to confirm these associations and move forward to the dietary guidelines and specific (poly)phenols recommendations in risk populations.

The present study has two main strengths. The first is the use of a new online analytical tool for the 24-HDR collection called “myfood24” in a recognized subcohort from Denmark. The second is the use of the most updated FCDB and standardized protocol and the in-house software for the (poly)phenol intake estimation. However, this study has a few weaknesses related to the well-known limitations of FCDBs in terms of incomplete data related to foods or (poly)phenols (analytical method or specific compounds), and the common measurement error of self-reported 24-HDR. Another limitation to be aware of is that some registered the same food item at different meal events. For example, coffee was mainly assigned to drink events; however, some participants classified it to other meal events (e.g. snack). This is very important to be considered and controlled in future studies. Finally, it would be optimally had three recalls from all participants to estimate the most valid mean intake, but that was only the case for 301 while 196 had two and 7 had one.

Future studies would consider the meal events and their dietary composition, food matrix, and processing. It will help researchers to investigate eating behavior, level of adherence and the diet bioavailability balance. Consuming most of the (poly)phenol-rich products between meals and/or skipping from the main meals could also impact their bioavailability, for example, the effect of using them as substrates for microbial transformation [32]. Another recommendation is the standardization of (poly)phenol dietary estimation, the FCDB used and the methods as express the (poly)phenols (i.e. glycosides vs aglycones) it’s very relevant for analyses, approaches, and perspectives that will proceed. Lastly, considering the challenges for inter-individuality and the potential metabotyping for diet (clusters or scores) [51], using metabolomics providing biomarkers would seem to have greater strength moving forward in terms of synergic analysis with diet questionnaires [52, 53].

In conclusion, this study provides the most detailed description of total (poly)phenol intake consumed in meal events in a MAX subcohort from Denmark. The meal events that provided the biggest contribution of (poly)phenols were drinks meal events, which occurred extensively during the day, and also the breakfast meal event. The main food sources for individual (poly)phenols were nonalcoholic beverages such as coffee and tea, cocoa products such as dark chocolate, and cereals such as rye products. The individual (poly)phenols consumed the most were hydroxycinnamic acids and proanthocyanidins. The alkylphenol consumption from cereal products is also remarkable. It would be also relevant to explore this predominance of (poly)phenol subclasses and their individual (poly)phenols in greater depth along with other outcomes such as health conditions. Moreover, we must consider sociodemographic and lifestyle factors since they are associated with differences in (poly)phenol intake. This descriptive analysis of total dietary (poly)phenols in meals provides key evidence that suggests the need for further investigation with improved approaches in epidemiological and clinical studies, thereby increasing the knowledge of the role of (poly)phenols in the prevention of diseases and in healthy aging.

## Supplementary Information

Below is the link to the electronic supplementary material.

Supplementary file1 (DOCX 31 KB)

## References

[1] Barba FJ, Esteve MJ, Frígola A (2014). Bioactive components from leaf vegetable products. Stud Nat Prod Chem.

[2] Li AN, Li S, Zhang YJ (2014). Resources and biological activities of natural polyphenols. Nutrients.

[3] Rothwell JA, Knaze V, Zamora-Ros R (2017). Polyphenols: dietary assessment and role in the prevention of cancers. Curr Opin Clin Nutr Metab Care.

[4] Zamora-ros R, Knaze V, Rothwell JA (2016). Dietary polyphenol intake in Europe : the European prospective investigation into cancer and nutrition (EPIC ) study. Eur J Nutr.

[5] Probst Y, Guan V, Kent K (2018). A systematic review of food composition tools used for determining dietary polyphenol intake in estimated intake studies. Food Chem.

[6] Scalbert A, Andres-Lacueva C, Arita M (2011). Databases on food phytochemicals and their health-promoting effects. J Agric Food Chem.

[7] Knaze V, Rothwell JA, Zamora-Ros R (2018). A new food-composition database for 437 polyphenols in 19,899 raw and prepared foods used to estimate polyphenol intakes in adults from 10 European countries. Am J Clin Nutr.

[8] Burkholder-cooley NM, Rajaram SS, Haddad EH (2017). Validating polyphenol intake estimates from a food-frequency questionnaire by using repeated 24-h dietary recalls and a unique method-of-triads approach with 2 biomarkers. Am J Clin Nutr.

[9] Zamora-Ros R, Biessy C, Rothwell JA (2018). Dietary polyphenol intake and their major food sources in the Mexican Teachers’ Cohort. Br J Nutr.

[10] Marranzano M, Ray S, Godos J, Galvano F (2018). Association between dietary flavonoids intake and obesity in a cohort of adults living in the Mediterranean area. Int J Food Sci Nutr.

[11] Bondonno NP, Dalgaard F, Kyrø C (2019). Flavonoid intake is associated with lower mortality in the Danish diet cancer and health cohort. Nat Commun.

[12] Del Bo C, Bernardi S, Marino M (2019). systematic review on polyphenol intake and health outcomes: is there sufficient evidence to define a health-promoting polyphenol-rich dietary pattern?. Nutrients.

[13] Hussain S, Bloom S (2013). The regulation of food intake by the gut-brain axis: implications for obesity. Int J Obes.

[14] Vandevijvere S, Mackay S, D’Souza E, Swinburn B (2019). The first INFORMAS national food environments and policies survey in New Zealand: a blueprint country profile for measuring progress on creating healthy food environments. Obes Rev.

[15] Monteiro CA, Cannon G, Levy RB (2019). Ultra-processed foods: What they are and how to identify them. Public Health Nutr.

[16] Young KG, Duncanson K, Burrows T (2018). Influence of grandparents on the dietary intake of their 2–12-year-old grandchildren: a systematic review. Nutr Diet.

[17] Touyz LM, Wakefield CE, Grech AM (2018). Parent-targeted home-based interventions for increasing fruit and vegetable intake in children: a systematic review and meta-analysis. Nutr Rev.

[18] Martini D, Bernardi S, Del Bo’ C (2020). Estimated intakes of nutrients and polyphenols in participants completing the maple randomised controlled trial and its relevance for the future development of dietary guidelines for the older subjects. Nutrients.

[19] Petersen K, Halkjær J, Steffen L (2022). Estimated intakes of nutrients and polyphenols in participants completing the maple randomised controlled trial and its relevance for the future development of dietary guidelines for the older subjects. Eur J Epidemiol.

[20] Tjønneland A, Olsen A, Boll K (2007). Study design, exposure variables, and socioeconomic determinants of participation in diet, cancer and health: a population-based prospective cohort study of 57,053 men and women in Denmark. Scand J Public Health.

[21] Wark PA, Hardie LJ, Frost GS (2018). Validity of an online 24-h recall tool (myfood24) for dietary assessment in population studies: Comparison with biomarkers and standard interviews. BMC Med.

[22] Vitale M, Masulli M, Rivellese AA (2018). Dietary intake and major food sources of polyphenols in people with type 2 diabetes: the TOSCA.IT study. Eur J Nutr.

[23] Tjønneland A, Overvad K, Haraldsdóttir J (1991). Validation of a semiquantitative food frequency questionnaire developed in Denmark. Int J Epidemiol.

[24] Institut National de la Recherche Agronomique (INRA) (2013) Phenol-Explorer 3.6: database on polyphenol content in foods. http://www.phenol-explorer.eu/

[25] de Lima KCM, de Barros HDF, Passos TS, Maciel BLL (2019). The effect of using different oils and paper towel in vegetable oil absorption of fried recipes. J Culin Sci Technol.

[26] Lanuza (2021). Advances in polyphenol research from Chile: a literature review. Food Rev Int.

[27] Pérez-Jiménez J, Fezeu L, Touvier M (2011). Dietary intake of 337 polyphenols in French adults. Am J Clin Nutr.

[28] Tresserra-Rimbau A, Medina-Remón A, Pérez-Jiménez J (2013). Dietary intake and major food sources of polyphenols in a Spanish population at high cardiovascular risk: the PREDIMED study. Nutr Metab Cardiovasc Dis.

[29] Witkowska AM, Zujko ME, Waśkiewicz A (2015). Comparison of various databases for estimation of dietary polyphenol intake in the population of polish adults. Nutrients.

[30] Miranda AM, Steluti J, Fisberg RM, Marchioni DM (2016). Dietary intake and food contributors of polyphenols in adults and elderly adults of Sao Paulo: a population-based study. Br J Nutr.

[31] Hinojosa-Nogueira D, Pérez-Burillo S, Garciá-Rincón I (2021). A useful and simple tool to evaluate and compare the intake of total dietary polyphenols in different populations. Public Health Nutr.

[32] Bohn T (2014). Dietary factors affecting polyphenol bioavailability. Nutr Rev.

[33] Zamora-Ros R, Knaze V, Rothwell JA (2016). Dietary polyphenol intake in Europe: the European prospective investigation into cancer and nutrition (EPIC) study. Eur J Nutr.

[34] Zamora-Ros R, Rothwell JA, Scalbert A (2013). Dietary intakes and food sources of phenolic acids in the European prospective investigation into cancer and nutrition (EPIC) study. Br J Nutr.

[35] Grosso G, Stepaniak U, Topor-Madry R (2014). Estimated dietary intake and major food sources of polyphenols in the Polish arm of the HAPIEE study. Nutrition.

[36] Zamora-Ros R, Knaze V, Romieu I (2013). Impact of thearubigins on the estimation of total dietary flavonoids in the European prospective investigation into cancer and nutrition (EPIC) study. Eur J Clin Nutr.

[37] Ivey KL, Croft K, Prince RL, Hodgson JM (2016). Comparison of flavonoid intake assessment methods. Food Funct.

[38] Ziauddeen N, Rosi A, Del Rio D (2019). Dietary intake of (poly)phenols in children and adults: cross-sectional analysis of UK national diet and nutrition survey rolling programme (2008–2014). Eur J Nutr.

[39] Neveu V, Perez-Jiménez J, Vos F (2010). Phenol-Explorer: an online comprehensive database on polyphenol contents in foods. Database (Oxford).

[40] Haytowitz D, Wu X, Bhagwat S (2018) USDA database for the proanthocyanidin content of selected foods, release 2.1. U.S. Departament of Agriculture Agricultural Service. Nutrient Data Laboratory Home Page: http://www.ars.usda.gov/nutrientdata/flav. Accessed 26 June 2022

[41] Andersen JLM, Halkjær J, Rostgaard-Hansen AL (2021). Intake of whole grain and associations with lifestyle and demographics: a cross-sectional study based on the Danish diet, cancer and health—next generations cohort. Eur J Nutr.

[42] Burkholder-Cooley N, Rajaram S, Haddad E (2016). Comparison of polyphenol intakes according to distinct dietary patterns and food sources in the Adventist health study-2 cohort. Br J Nutr.

[43] Pérez-Jiménez J, Neveu V, Vos F, Scalbert A (2010). Identification of the 100 richest dietary sources of polyphenols: an application of the Phenol-Explorer database. Eur J Clin Nutr.

[44] Wisnuwardani RW, De Henauw S, Androutsos O (2019). Estimated dietary intake of polyphenols in European adolescents: the HELENA study. Eur J Nutr.

[45] Taguchi C, Fukushima Y, Kishimoto Y (2015). Polyphenol intake from beverages in Japan over an 18-Year period (1996–2013): trends by year, age, gender and season. J Nutr Sci Vitaminol (Tokyo).

[46] Taguchi C, Kishimoto Y, Takeuchi I (2019). Estimated dietary polyphenol intake and its seasonal variations among Japanese university students. J Nutr Sci Vitaminol (Tokyo).

[47] Kent K, Charlton KE, Lee S (2018). Dietary flavonoid intake in older adults: how many days of dietary assessment are required and what is the impact of seasonality?. Nutr J.

[48] Dalgaard F, Bondonno NP, Murray K (2019). Associations between habitual flavonoid intake and hospital admissions for atherosclerotic cardiovascular disease: a prospective cohort study. Lancet Planet Heal.

[49] Adriouch S, Kesse-Guyot E, Feuillet T (2018). Total and specific dietary polyphenol intakes and 6-year anthropometric changes in a middle-aged general population cohort. Int J Obes.

[50] Tresserra-Rimbau A, Castro-Barquero S, Vitelli-Storelli F (2019). Associations between dietary polyphenols and type 2 diabetes in a cross-sectional analysis of the PREDIMED-Plus trial: role of body mass index and sex. Antioxidants.

[51] Posma JM, Garcia-Perez I, Frost G (2020). Nutriome–metabolome relationships provide insights into dietary intake and metabolism. Nat Food.

[52] Palmnäs M, Brunius C, Shi L (2019). Perspective: metabotyping—a potential personalized nutrition strategy for precision prevention of cardiometabolic disease. Adv Nutr.

[53] Ruskovska T, Maksimova V, Milenkovic D (2020). Polyphenols in human nutrition: from the in vitro antioxidant capacity to the beneficial effects on cardiometabolic health and related inter-individual variability: an overview and perspective. Br J Nutr.

