# Supplementary material for: Descriptive analysis of dietary (poly)phenol intake in the subcohort MAX from DCH-NG: “Diet, Cancer and Health—Next Generations cohort”
Source: Eur J Nutr. 2022 Aug 22;62(1):337–50. doi: 10.1007/s00394-022-02977-x (PMC9899735; doi:10.1007/s00394-022-02977-x)
Supplement: Supplementary file 1 — Supplementary file1 (DOCX 31 KB) [file 394_2022_2977_MOESM1_ESM.docx]

Supplementary Table 1. **Nonadjusted mean daily intakes of total and (poly)phenols meal event by sex, age and lifestyle factors in MAX subcohort.**

| **Stratification Variable** | **N** | **All**  **(mg/day)** | | | **Breakfast**  **(mg/day)** | | | **Lunch**  **(mg/day)** | | | **Evening**  **(mg/day)** | | | **Snack**  **(mg/day)** | | | **Drink**  **(mg/day)** | | |
| --- | --- | --- | --- | --- | --- | --- | --- | --- | --- | --- | --- | --- | --- | --- | --- | --- | --- | --- | --- |
|  |  | **Mean** | **s.e** | **p value** | **Mean** | **s.e** | **p value** | **Mean** | **s.e** | **p value** | **Mean** | **s.e** | **p value** | **Mean** | **s.e** | **p value** | **Mean** | **s.e** | **p value** |
| **Total (poly)phenol** | 676 | 1325 | 34 |  | 293 | 12 |  | 159 | 7 |  | 141 | 5 |  | 245 | 11 |  | 486 | 26 |  |
| **Time origin** |  |  |  | 0.285 |  |  | 0.306 |  |  | 0.794 |  |  | 0.264 |  |  | 0.050 |  |  | 0.198 |
| Baseline | 648 | 1371 | 41 |  | 309 | 16 |  | 178 | 9 |  | 143 | 7 |  | 310 | 18 |  | 823 | 49 |  |
| 6 months | 406 | 1316 | 52 |  | 350 | 20 |  | 168 | 11 |  | 161 | 9 |  | 297 | 23 |  | 719 | 64 |  |
| 12 months | 382 | 1436 | 53 |  | 329 | 21 |  | 177 | 11 |  | 144 | 9 |  | 374 | 24 |  | 881 | 65 |  |
| **Sex** |  |  |  | 0.002 |  |  | 0.934 |  |  | 0.336 |  |  | 0.038 |  |  | 0.050 |  |  | < 0.001 |
| Men | 305 | 1440 | 50 |  | 294 | 18 |  | 166 | 10 |  | 154 | 8 |  | 219 | 17 |  | 588 | 38 |  |
| Women | 371 | 1231 | 45 |  | 292 | 17 |  | 153 | 9 |  | 130 | 7 |  | 266 | 15 |  | 402 | 35 |  |
| **Age (y)** |  |  |  | < 0.001 |  |  | 0.004 |  |  | 0.149 |  |  | 0.562 |  |  | 0.440 |  |  | < 0.001 |
| 18–34 | 197 | 1034 | 61 |  | 228 | 23 |  | 143 | 13 |  | 141 | 10 |  | 222 | 21 |  | 297 | 47 |  |
| 35–50 | 210 | 1359 | 59 |  | 309 | 22 |  | 178 | 12 |  | 133 | 8 |  | 251 | 20 |  | 486 | 46 |  |
| >50 | 269 | 1512 | 52 |  | 327 | 19 |  | 155 | 11 |  | 147 | 8 |  | 256 | 18 |  | 625 | 41 |  |
| **Body mass index (kg/m2)** |  |  |  | 0.008 |  |  | 0.203 |  |  | 0.065 |  |  | 0.776 |  |  | 0.607 |  |  | < 0.001 |
| <25 | 371 | 1252 | 45 |  | 299 | 16 |  | 159 | 9 |  | 138 | 7 |  | 255 | 15 |  | 398 | 35 |  |
| 25 to 30 | 236 | 1360 | 57 |  | 268 | 21 |  | 145 | 11 |  | 146 | 9 |  | 233 | 19 |  | 567 | 44 |  |
| >30 | 69 | 1599 | 106 |  | 343 | 39 |  | 203 | 22 |  | 137 | 17 |  | 230 | 36 |  | 684 | 81 |  |
| **Smoking status** |  |  |  | 0.039 |  |  | 0.408 |  |  | 0.606 |  |  | 0.467 |  |  | 0.640 |  |  | 0.009 |
| Never smoker | 353 | 1243 | 47 |  | 278 | 17 |  | 152 | 9 |  | 147 | 7 |  | 255 | 16 |  | 409 | 36 |  |
| Former smoker | 186 | 1425 | 64 |  | 317 | 23 |  | 166 | 13 |  | 139 | 10 |  | 232 | 22 |  | 569 | 49 |  |
| Current smoker | 137 | 1401 | 75 |  | 297 | 27 |  | 167 | 15 |  | 129 | 12 |  | 235 | 25 |  | 572 | 58 |  |
| **Physical activity** |  |  |  | 0.185 |  |  | 0.683 |  |  | 0.581 |  |  | 0.542 |  |  | 0.676 |  |  | 0.154 |
| Regular | 562 | 1305 | 37 |  | 290 | 13 |  | 157 | 7 |  | 139 | 6 |  | 247 | 12 |  | 469 | 28 |  |
| Not regular | 114 | 1426 | 82 |  | 304 | 30 |  | 167 | 17 |  | 149 | 13 |  | 234 | 28 |  | 570 | 64 |  |
| **Energy intake (kcal)** |  |  |  | < 0.001 |  |  | 0.002 |  |  | <0.001 |  |  | < 0.001 |  |  | <0.001 |  |  | 0.111 |
| Tertile 1 | 225 | 1032 | 56 |  | 238 | 21 |  | 126 | 12 |  | 113 | 9 |  | 140 | 19 |  | 414 | 45 |  |
| Tertile 2 | 226 | 1310 | 56 |  | 296 | 21 |  | 152 | 12 |  | 136 | 9 |  | 228 | 19 |  | 497 | 45 |  |
| Tertile 3 | 225 | 1634 | 56 |  | 345 | 21 |  | 198 | 12 |  | 174 | 9 |  | 367 | 19 |  | 548 | 45 |  |
| **Alcohol intake (g/d)** |  |  |  | < 0.001 |  |  | 0.015 |  |  | 0.148 |  |  | 0.060 |  |  | 0.051 |  |  | 0.054 |
| No | 339 | 1201 | 47 |  | 262 | 17 |  | 149 | 9 |  | 130 | 7 |  | 222 | 16 |  | 436 | 37 |  |
| Yes | 337 | 1450 | 47 |  | 323 | 17 |  | 169 | 10 |  | 152 | 7 |  | 267 | 16 |  | 537 | 37 |  |

Supplementary Table 2. **Nonadjusted mean daily intakes of total and (poly)phenols classes by season in MAX subcohort**^1^**.**

|  | All  (n= 676) | Season | | | | |
| --- | --- | --- | --- | --- | --- | --- |
|  |  | Fall | Winter | Spring | Summer | p-value |
| **Participants**  (k measures) | 1392^2^ | 555 | 272 | 187 | 378 |  |
| **(Poly)phenols** |  |  |  |  |  |  |
| Mean ± SE | 1325 ± 34 | 1354 ± 44 | 1310 ± 64 | 1444 ± 77 | 1426 ± 54 | 0.406 |
| Median  (p-25-p75) | 1164  (722-1731) | 1119  (678-1762) | 1095  (619-1743) | 1219  (747-1865) | 1235  (673-1863) |  |
| **Flavonoids** |  |  |  |  |  |  |
| Mean ± SD | 427 ± 11 | 412 ± 18 | 445 ± 25 | 434 ± 31 | 446 ± 21 | 0.581 |
| Median  (p-25-p75) | 312  (141-592) | 302  (144-585) | 349  (125-603) | 306  (137-573) | 328  (152-627) |  |
| **Phenolic acids** |  |  |  |  |  |  |
| Mean ± SD | 868 ± 25 | 864 ± 40 | 791 ± 58 | 930 ± 70 | 900 ± 49 | 0.395 |
| Median  (p-25-p75) | 598  (255-1178) | 614  (262-1199) | 520  (198-1060) | 641  (350-1252) | 575  (277-1219) |  |
| **Stilbenes** |  |  |  |  |  |  |
| Mean ± SD | 1.7 ± 0.1 | 1.6 ± 0.1 | 1.7 ± 0.2 | 1.5 ± 0.3 | 1.7 ± 0.2 | 0.964 |
| Median  (p-25-p75) | 0.01  (0.00-0.50) | 0.01  (0.00-0.43) | 0.01  (0.00-0.78) | 0.02  (0.00-1.12) | 0.01  (0.00-0.53) |  |
| **Lignans** |  |  |  |  |  |  |
| Mean ± SD | 11 ± 0.5 | 8.5 ± 0.8 | 7.4 ± 1.1 | 6.5 ± 1.3 | 8.7 ± 1.0 | 0.510 |
| Median  (p-25-p75) | 2.0  (0.6-17.4) | 2.0  (0.6-17.6) | 2.1  (0.6-16.5) | 3.1  (0.8-19.8) | 1.5  (0.6-16.0) |  |
| **Alkylphenols** |  |  |  |  |  |  |
| Mean ± SD | 39 ± 1.0 | 38.5 ± 1.6 | 38.2 ± 2.3 | 42.4 ± 2.8 | 38.9 ± 2.0 | 0.653 |
| Median  (p-25-p75) | 28.1  (9.7-53.5) | 28.8  (9.4-53.2) | 28.1  (9.1-52.7) | 28.6  (9.7-57.6) | 28.1  (11.1-54.0) |  |
| **Tyrosols** |  |  |  |  |  |  |
| Mean ± SD | 11 ± 0.4 | 11.5 ± 0.7 | 11.0 ± 1.0 | 9.8 ± 1.2 | 12.5 ± 0.9 | 0.380 |
| Median  (p-25-p75) | 6.9  (2.3-14.4) | 7.0  (2.2-13.8) | 8.0  (2.5-15.2) | 6.1  (2.3-14.1) | 6.8  (2.3-15.2) |  |

^1^Means and standard error (s.e.) were computed using general linear models. P value is for differences in means. ^2^1392 of 1436 total participants had a season date register.
